# In Silico Screening of Bioactive Peptides in Stout Beer and Analysis of ACE Inhibitory Activity

**DOI:** 10.3390/foods13131973

**Published:** 2024-06-22

**Authors:** Wenhui Tian, Cui Zhang, Qi Zheng, Shumin Hu, Weiqiang Yan, Ling Yue, Zhijun Chen, Ci Zhang, Qiulian Kong, Liping Sun

**Affiliations:** 1 Crop Breeding and Cultivation Research Institute, Shanghai Academy of Agricultural Sciences, Shanghai 201403, China; twh9768@163.com (W.T.); zhengqi@saas.sh.cn (Q.Z.); webweifree@163.com (W.Y.); yueling312@sina.com (L.Y.); cizhang6@saas.sh.cn (C.Z.); 2 State Key Laboratory of Biological Fermentation Engineering of Beer, Qingdao 266021, China; zhangcui@tsingtao.com.cn (C.Z.); hsm@tsingtao.com.cn (S.H.); 3 Shanghai Shuneng Irradiation Technology Co., Ltd., Shanghai Academy of Agricultural Sciences, Shanghai 201403, China; 13764023962@126.com; 4 Faculty of Food Science and Engineering, Kunming University of Science and Technology, Kunming 650500, China

**Keywords:** beer polypeptide, angiotensin converting enzyme, virtual screening, molecular docking

## Abstract

Stout beer was selected as the research object to screen angiotensin-converting enzyme (ACE) inhibitory peptides. The peptide sequences of stout beer were identified using ultra-performance liquid chromatography-quadrupole-Orbitrap mass spectrometry with de novo, and 41 peptides were identified with high confidence. Peptide Ranker was used to score the biological activity and six peptides with a score ≥ 0.5 were screened to predict their potential ACE inhibitory (ACEI) activity. The toxicity, hydrophilicity, absorption, and excretion of these peptides were predicted. In addition, molecular docking between the peptides and ACE revealed a significant property of the peptide DLGGFFGFQR. Furthermore, molecular docking conformation and molecular dynamics simulation revealed that DLGGFFGFQR could be tightly bound to ACE through hydrogen bonding and hydrophobic interaction. Lastly, the ACEI activity of DLGGFFGFQR was confirmed using in vitro evaluation and the IC_50_ value was determined to be 24.45 μM.

## 1. Introduction

Hypertension is one of the most common chronic diseases with the highest incidence rate and it is also an important risk factor for cardiovascular disease (CVDs) [1,2]. On 19 September 2023, the World Health Organization published its inaugural Global Hypertension Report, which stated that the prevalence of hypertension had steadily increased over the past 2–3 decades, with the number of adults with hypertension doubling from 650 million in 1990 to 1.3 billion in 2019 [3]. The angiotensin-converting enzyme (ACE) plays a pivotal role in the renin-angiotensin system. ACE cleaves the C-terminal histidine and leucine residues of the decapeptide angiotensin I, transforming it into the effective vasoconstrictor angiotensin II and causing an increase in blood pressure [4,5]. Therefore, hypertension can be controlled by inhibiting ACE activity. Food-derived ACE inhibitory (ACEI) peptides are a class of peptides with antihypertensive activity. Unlike traditional antihypertensive drugs, they are non-toxic, have no adverse effects, and do not influence normal blood pressure [6,7]. They have the potential to replace antihypertensive drugs; therefore, the exploration of food-derived ACEI peptides is gradually becoming a research focus [8].

Traditional methods to identify peptide components involve the combination of ultrafiltration separation with purification methods such as volume-exclusion chromatography and ion-exchange chromatography [9]. These techniques can be used to isolate and characterize the individual components that have high activity. However, this method is not only challenging to perform but also provides inefficient results, limiting the number of peptides that can be obtained at a given time. Additionally, there is a risk of losing some highly active components during separation, which hinders high-throughput screening efforts. Proteomics and mass spectrometry techniques have advanced significantly. In silico screening eliminates the need for tedious separation and purification processes and allows batch processing of many polypeptides via rapid screening. A combination of bioinformatics and online databases can be used to screen several peptide sequences for potential bioactivity. This method is rapid, highly efficient and inexpensive, making it especially suitable for screening ACEI peptides [10].

Recently, several ACEI peptides have been identified and evaluated in brewed wine. Wu et al. discovered a polypeptide in Chinese Baijiu for the first time, which indicated an ACE inhibitory effect with an IC_50_ of 446 mmol/L [11]. Guo et al. confirmed the potential antioxidant and active ACEI peptides isolated from Guangdong glutinous rice wine and found significantly increased ACEI activity (30.89%) [12]. Moreover, Yin et al. identified two peptides, LPVGP and FPLQPHQP, from Qingke Baijiu, which had an inhibition with ACE (IC_50_ = 9.05, 5.03 μM) [13]. Beer is one of the most globally consumed fermented beverages that traditionally comprises four ingredients, including malted barley, water, hops, and yeast [14,15]. It is rich in nutrients, has a low alcohol content and can dissolve various peptides, amino acids, vitamins, and some enzymes (either raw or in the process) [16]. Numerous epidemiological studies have reported the protective effects of beer against CVDs [17]. A study reported that light-to-moderate beer drinkers were 57% less likely to develop CVDs than abstainers [18]. Ronan et al. reported that ale, lager, and stout could significantly inhibit platelet-activating factor-induced human platelet aggregation in vitro [19]. It has been observed that beer contains 2–6 mg/mL of protein/peptide [20], primarily derived from the water-soluble protein and insoluble barley protein in the raw material; some peptides are also produced by yeast metabolism during fermentation [21]. However, there has been little research on bioactive peptides in beer.

Stout beer, also known as dark beer, is generally black or brown in color and is prepared using special malts such as burnt and black malt as brewing materials [22,23]. This beer has unique malt and burnt-malt flavors, offering a mellow and slightly sweet taste [24]. Stout beer is rich in nutrients and its amino acid content is 3–4 times higher than that in other types of beers [25]. This study aims to identify peptides in stout beer, screen for bioactive peptides with ACEI activity through in silico screening and verify the activities of the identified peptides in vitro. Furthermore, molecular docking and molecular dynamics simulation (MD) were used to study the interaction between the peptide and ACE. This study could serve as a valuable reference for the development of beer with certain bioactivity.

## 2. Materials and Methods

### 2.1. Materials and Reagents

Stout beer was purchased from a market in Kunming, China. Synthesized peptides were obtained from Shanghai Synpeptide Co., Ltd., (Shanghai, China). An ACE activity determination kit was obtained from Sigma-Aldrich (Saint Louis, MO, USA). Trifluoroacetic acid and acetonitrile were purchased from Merck (Darmstadt, Germany).

### 2.2. Sample Preparation

The stout beer samples were filtered, degassed, and then concentrated from 300 mL to 50 mL. After concentration, the samples were extracted three times with ethyl acetate [26]. The aqueous phase obtained after extraction was subsequently used for peptide analysis.

### 2.3. Isolation and Identification of Peptides

The peptides in stout beer were analyzed using ultra-performance liquid chromatography-quadrupole-Orbitrap mass spectrometry (UPLC-Q-Orbitrap-MS^2^) [27].

The UPLC conditions were as follows: InfinityLab Poroshell 120 EC-C18 (2.1 × 100 mm, 1.9 μm); column temperature of 30 °C; mobile phase A: acetonitrile with 0.1% formic acid; mobile phase B: ultrapure water with 0.1% formic acid. The injection volume was 2 μL and the flow rate was 0.2 mL/min. Gradient elution was 5% A from 0 to 1 min, 10% A from 1 to 2.5 min, 25% A from 2.5 to 12.5 min, 52.5% A from 12.5 to 20 min, 95% A from 20 to 22 min, and 5% A from 22 to 30 min.

The following conditions were used for mass spectrometry: ESI+ ion source; the primary mass spectrometer resolution was 70,000, and the secondary mass spectrum was 35,000; the spray voltage was 3.2 kV; the capillary temperature was 350 °C; the drier temperature was 350 °C; the data acquisition range was m/z 100–1000; the mass spectrometry data scanning mode was full MS-dd MS^2^.

The obtained mass data were analyzed using Peaks Studio 8.0 software. Only peptides with an average local confidence (ALC) score ≥ 85% were considered for further analysis.

### 2.4. Screening of Bioactive Peptides In Silico

In silico digestion was performed to compare the selected peptides with known ACEI peptides in the BIOPEP database (http://www.uwm.edu.pl/biochemia/index.php/en/biopep) (last accessed on 4 April 2024) and the AHTPDB database (http://crdd.osdd.net/raghava/ahtpdb/) (last accessed on 4 April 2024).

Peptide Ranker (http://bioware.ucd.ie/compass/biowareweb/Serverpages/Peptideranker) (last accessed on 8 April 2024) was utilized to predict the possible bioactivities of the peptides. Peptides with a score ≥ 0.5 indicated potential biological activity [28]. In this study, a threshold of 0.5 was established for activity evaluation.

The BIOPEP application (http://www.uwm.edu.pl/Biochemia/biopep/start_biopep.php) (last accessed on 10 April 2024) contains a bioactive peptide sequence database and a program. The program can be used to construct potential bioactivity profiles of peptides, calculating quantitative descriptors to measure their potential values. In this study, the “Activity” and “Profiles of potential biological activity” functions were used to search for potential biological activity and possible active sites.

### 2.5. Toxicity and Solubility Prediction

ToxinPred (https://webs.iiitd.edu.in/raghava/toxinpred/) (last accessed on 12 April 2024) is a unique and valuable in silico method used to predict the toxicity of peptides and proteins. The Expasy database (https://web.expasy.org/protparam/) (last accessed on 14 April 2024) was used to calculate the hydrophilicity and hydrophobicity indices of the amino acids in peptides, which were assessed and represented using the grand average of hydropathy value (GRAVY). A low GRAVY value indicates strong hydrophilicity, which is conducive to solubility, and vice versa [29].

### 2.6. Absorption and Excretion Properties Analysis

The prediction of absorption and excretion properties is essential in contemporary drug design and screening. In this study, AdmetSAR (http://lmmd.ecust.edu.cn/admetsar1) (last accessed on 15 April 2024) was used to predict the properties of peptides. Furthermore, the primary evaluation indicators selected in this study indicated the blood–brain barrier (BBB), human intestinal absorption (HIA), and cytochrome P450 inhibitory promiscuity (CYP 450) [30]. The peptide codes were translated into simplified molecular input line entry specifications using PepSMI (https://www.novoprolabs.com/tools) (last accessed on 18 April 2024).

### 2.7. Molecular Docking

#### 2.7.1. Preparation of Small Molecules

Peptide segment structures were built using the “Build Protein” function in SYBYL-X 2.1.1 software, followed by optimization with parameters set to Max Iterations 5000 and Gradient 0.005 to ensure energy minimization [31].

#### 2.7.2. Preparation of Protein Structure

The crystal structure of ACE (PDBI: 108A) was downloaded from Protein Data Bank (http://www.rcsb.org/pdb) (last accessed on 20 April 2024). The enzyme was processed using the “Surflex-Dock” function in SYBYL-X. The ligand molecule from the original receptor-ligand complex crystal structure was separated from the receptor pocket, water molecules and other small molecules were removed, and the protein was hydrogenated and charged. A docking interface was set within 5 Å of the ligand center.

#### 2.7.3. Molecular Docking

The peptide segments were flexibly docked with ACE using the “Surflex-dock” function in SYBYL-X. Peptides were docked into ACE using molecular visualization. The total score (T-Score), consensus score (C-Score), and number of hydrogen bonds were used to evaluate the docking results. A C-Score ≥ 4.0 indicated successful docking, and a T-Score ≥ 6.0 indicated superior docking [32]. Based on the evaluation criteria, optimal conformation was selected.

#### 2.7.4. 2D Structure Visualization

The LigPlot program was used to analyze the 2D interaction of the receptor-ligand complex, the interaction between proteins and the ligand complex was determined, and the hydrophobic bond, hydrogen bond and bond length under docking posture were analyzed.

### 2.8. MD Simulation

The simulation of the peptides and the ACE was performed according to a previous report [33,34]. In the preprocessing of small molecules, Gaussian 16 W was used for the hydrogenation of small molecules, whereas AmberTools 22 was used to impose GAFF on the small molecule force field. Amber99sb-ildn was used as the force field, and a TIP3P water model as the solvent. Na+ ions were used to neutralize the charge. The simulation system adopts the steepest descent method to minimize the energy; the coupling constant was 0.1 ps and the duration was 100 ps. The following conditions were employed: temperature: 300 K, pressure: 1 atm, time: 100 ns, isothermal and isobaric system. The root-mean-square deviation (RMSD), root-mean-square fluctuation (RMSF), the solvent-accessible surface area (SASA), changes in the radius of gyration, and the number of hydrogen bond changes during MD simulation were determined.

### 2.9. Determination of ACEI Activity In Vitro

ACEI activity determination was determined using an assay kit. Briefly, 10 μL of ACE and 40 μL of the inhibitor were added to the microplate and mixed thoroughly, then 50 μL of the substrate was added to start the reaction. The fluorescence was detected at an excitation wavelength of 320 nm and an emission wavelength of 405 nm at 37 °C for 30 min. The ACE activity of the peptide was calculated as follows:

ACEI activity = [(K_Std_ − K_Sample_)/K_Std_] × DF × 100%

where K_Sample_ means the slope of the sample curve after subtracting the blank. (RFU/min); K_Std_ means the slope of the standard curve after subtracting the blank (RFU/min); DF means the dilution factor.

### 2.10. Statistical Analysis

Each experiment was conducted in triplicate, the data were presented as mean ± standard deviation. SYBYL-X 2.1.1 software was utilized for evaluating the molecular docking visualization. LigPlot 1.4.5 software was employed to illustrate the 2D interactions. MD simulation data analysis was performed using GROMACS 2022.3 software.

## 3. Results and Discussion

### 3.1. Identification of Peptides

During the brewing process, most proteins are enzymatically metabolized into small molecule polypeptides or single amino acids and are ultimately found in the final beer product. Previous studies were primarily focused on the proteomic composition of beer. Mahya et al. have reviewed the types of proteins in beer [35]. Gianluca and co-authors analyzed the protein and peptide components of two Italian barley malt beers and indicated that Z4-barley protein, ns-LTP1 and two barley albumins are predominantly found in beer [36,37].

In this study, peptides in stout beer were identified using UPLC-Q-Orbitrap-MS^2^, and a total of 41 peptides with ALC ≥ 85% were selected (Table 1). The peptides PMAPLPRSGPE and DLGGFFGFQR from Italian beer, LPQQQAQFK, STEWHLD, PPPVHD, LVLPGELAK, LAVMQQQQQQ, PPPVHDTD, PPVPHDTD, LPEDAKVE, HAVSEGTKAVT, LPGELAK, PMAPLPRSGEP, TVSGF, LNFDPNR, LALDTRVG, LDTRVGV, and RVALVY from draft beer, which were previously identified, also appeared in our results [37,38]. The polypeptides present in beer primarily originate from malt protein. Previous studies have shown that the soluble protein content in malt water-soluble protein decreased significantly with an increase in roasting degree [39]. The main raw material of stout beer includes roasted burnt malt, and because roasted stout beer has fewer peptides than other beers [40]. The 41 peptides consisted of 4–14 amino acids, with a molecular weight less than 1600 Da, consistent with the structure of bioactive peptides [41]. Malt typically contains 8–14% protein, and is composed primarily of 18 different amino acids, of which Glu (2.9%), Pro (1.2%) and Leu (1.0%) are the most abundant [38]. Among the 41 peptides identified in this study, 18 contained Glu, 17 contained Pro, and 28 contained Leu, which is consistent with findings from previous studies. Moreover, the obtained peptides were entered into the active peptide database BIOPEP and ACEI peptide database AHTPDB and it was revealed that two of them were the already known peptides LNFDPNR and LPQQQAQFK (IC50 = 38.58, 26.45 µM) [42]. Therefore, the remaining 39 peptides were selected for the current study.

### 3.2. Process of Selecting Stout Beer Peptides

#### 3.2.1. Activity Score and Prediction

Peptide Ranker is a neural-network-based biopeptide activity prediction server. For each peptide, the score is between 0 and 1 [42]. The higher the peptide ranker score, the greater the possibility of biological activity. The Peptide Ranker scores of 39 peptides are shown in Table 2. The activity evaluation threshold was set at 0.5, based on which six peptides were screened, including DLGGFFGFQR, PMAPLPR, SSLF, PPPVHDFMNE, FDRLQ, and PMAPLPRSGEP with scores of 0.8736, 0.8565, 0.8145, 0.7050, 0.5909, 0.5780, respectively.

The potential biological activities of six peptides were predicted using the “Activity” function of BIOPEP, and the results showed that all peptides were potential ACE inhibitors. The active sites of the selected peptides were predicted using the function “Profiles of potential biological activity” and the results were as follows: GF, FG, GG, LG, FQ in peptide DLGGFFGFQR; PLP, PR, PL, AP, MAP in peptide PMAPLPR; LF in peptide SSLF; PP, DF, PPP in peptide PPPVHDFMNE; RL, LQ in peptide FDRLQ; PLP, PR, PL, GEP, AP, GE, SG, MAP in peptide PMAPLPRSGEP (Table 3). A previous study found that peptides containing Tyr, His, Phe, Arg, and Trp are characteristic of ACEI peptides [43]. The six peptides screened by BIOPEP exhibited these characteristics.

#### 3.2.2. Evaluation of the Properties of the Selected Peptides

Currently, peptides are being used to treat diseases; however, peptide-based therapy is limited by its toxicity profile. Therefore, it is necessary to predict the toxicity of polypeptides before commencing in-depth studies. ToxinPred is a useful tool to distinguish toxic peptides from the non-toxic ones. Here, the screen peptides were non-toxic, indicating their suitability as food-derived ACEI peptides (Table 4).

Good water solubility is the basis of orally bioactive peptides. The physiological activity of bioactive peptides is closely related to the hydrophobic/hydrophilic properties of the amino acid chains. The GRAVY value reflects the solubility of the peptides and ranges from −2 to 2. Positive values indicate that the peptide is hydrophobic, and negative values indicate hydrophilicity. The GRAVY value of SSLF was 1.250 and that of the other five peptides ranged from −0.050 to −0.980, indicating that SSLF is hydrophobic, whereas DLGGFFGFQR, PMAPLPR, SSLF, PPPVHDFMNE, FDRLQ, PMAPLPRSGEP are hydrophilic.

Absorption and excretion parameters play a crucial role in the identification of food-derived bioactive peptides [44]. Therefore, the properties of the six identified peptides were assessed using the AdmetSAR program (Table 4). The ability of chemicals to cross the BBB is important for the development of central nervous system drugs. Here, it was observed that all six peptides had positive BBB values, suggesting their BBB-penetrating potential and ability to enter the central nervous system. HIA results showed that DLGGFFGFQR, SSLF, PPPVHDFMNE and FDRLQ were HIA+, indicating that they have favorable permeability and absorptive properties. The HIA values of PMAPLPR and PMAPLPRSGEP were negative, indicating that they were not well absorbed after oral administration. CYP 450 is one of the most important enzymes in drug metabolism. Several CYP450 enzymes are involved in the metabolism of various endogenous and exogenous compounds, including active peptides and other dietary products. We analyzed the interactions between the selected peptides and CYP 450 enzymes to determine their metabolic characteristics. The results revealed that CYP 450 enzymes do not affect the efficacy and safety of peptides. Therefore, based on the evaluation of the properties of peptides, we selected three peptides DLGGFFGFQR, PPPVHDFMNE, FDRLQ for further analyses.

### 3.3. Molecular Docking

Molecular docking is widely used to study molecular interactions and predict binding patterns and forces. To further identify peptides with high ACEI activity, molecular docking was used to simulate and assess the binding capacity of the three peptides with ACE (Table 5). All three peptides had good binding ability with ACE, and the T-Score of DLGGFFGFQR was ≥10. Combining the characteristic findings from the peptide property analysis and the molecular docking score, DLGGFFGFQR was selected from among the three peptides owing to its strong binding ability to ACE.

### 3.4. Molecular Docking Conformation Analysis

**Figure 1:**
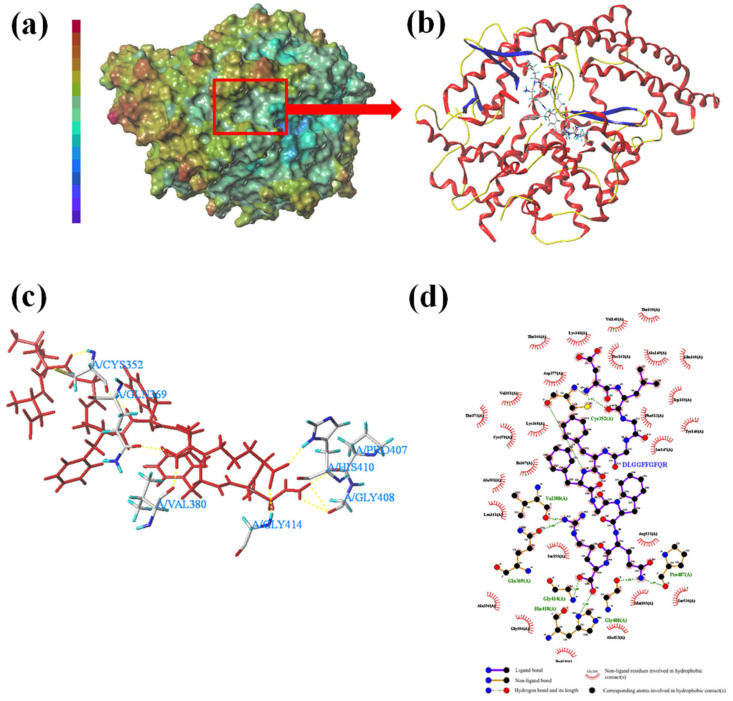
Docking result of DLGGFFGFQR-ACE. (**a**): ligand-protein molecular surface; (**b**): 3D structure of DLGGFFGFQR-ACE; (**c**): 3D binding interactions; (**d**): 2D binding interactions.

Molecular docking is a computational chemistry method that aims to predict binding patterns and affinity between proteins and small molecules. To understand the interactions between ACE and the peptides, DLGGFFGFQR was docked into the binding site of ACE (Table 6). The ACE active center includes 12 amino acid residues, which were Ala354, Gln281, Glu162, Glu384, Glu411, His353, His383, His387, His513, Lys511, Tyr520, and Tyr523 [45]. The ACE- DLGGFFGFQR binding site complex was predicted by SYBYL-X and the best conformations are shown in Figure 1. Figure 1a shows the surface of the ligand-protein molecule. Figure 1b is a 3D structure model of ACE-DLGGFFGFQR, whereas Figure 1c,d are the 3D and 2D models of the amino acid residue interaction binding mode. It was observed that DLGGFFGFQR formed eight hydrogen bonds with amino acids Cys352, Gln369, Val380, Pro407, His410, Gly408, and Gly414 in ACE, and the residues may enhance the ability of ACE to immobilize peptides and enhance stability, which may lead to increased inhibition. Based on relevant experimental reports and the findings related to the catalytic mechanism of ACE, it could be inferred that the results were similar to those for hemorphins [46]. Furthermore, DLGGFFGFQR formed 27 hydrophobic interactions with ACE and the action sites exhibited were different. Previous research showed that caffeic acid interacts with Asp377, Thr372, Thr166, Cys370, Pro163 at the ACE active site [47], corroborating our observations. This similarity suggested that the peptide was more tightly bound to ACE and may therefore be a more potent inhibitor.

### 3.5. MD Simulation Analysis

**Figure 2:**
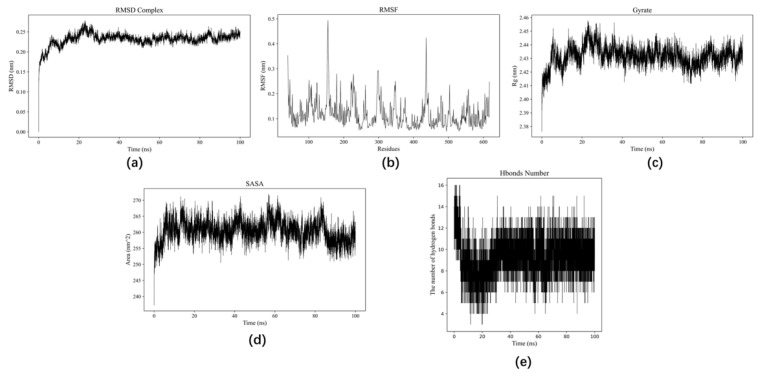
Results of MD simulation for the complexes of DLGGFFGFQR and ACE. (**a**): RMSD changes; (**b**): RMSF changes; (**c**): the radius of gyration changes; (**d**): SASA changes; (**e**): the number of hydrogen bond changes.

MD simulation indicated the interactions between the molecules at the atomic level of microscopic changes for dynamic analysis. The results of MD simulation for the complexes of DLGGFFGFQR and ACE are shown in Figure 2. The change in trend of RMSD of protein and ligand is an important characteristic indicating whether the simulation has reached a state of stability. The effect of DLGGFFGFQR-ACE receptor was more obvious between 0–40 ns and RMSD fluctuated widely, interfering with the overall structure. After 40 ns, RMSD was more stable around 2.5 Å. (Figure 2a).

RMSF is the mean value of the atomic position changes over time and can be used to characterize the flexibility and motion intensity of protein and amino acids during the simulation process. Amino acid residues with high values of RMSF have high variability and are also the center of the receptor structure region [48]. The amino acid residues in the 130–200 region were significantly affected by the interaction between DLGGFFGFQR and ACE (Figure 2b). At the same time, the RMSF value of this region was significantly higher than that of other regions, indicating that it may be an important area for DLG GFFGFQR to affect ACE and that amino acid fluctuations in this region would affect protein–peptide interactions. The molecular docking result showed that DLGGFFGFQR could form hydrophobic interactions with ACE via amino acid residues including Thr166, Val148, Thr150, Pro163, Ala149, Gln160, Trp185, Ser147, Tyr146, and Leu161. These two findings were in concordance.

The radius of gyration was used to characterize protein structure density. No significant changes in the radius of gyration were noted after the binding of the protein to the peptide (Figure 2c). SASA is used to assess the surface area of a protein molecule exposed to the solution and assess the interactions between them and the stability of the protein structure. The SASA of the ACE receptor decreased after binding (Figure 2d). Hydrogen bonding and hydrophobic interactions play an important role in preserving protein conformation. Figure 2e shows the changes in the number of hydrogen bonds, which tend to be stable after 40 ns.

Based on the above results, it could be concluded that DLGGFFGFQR and ACE had stable interactions. Moreover, the binding of the peptide caused protein compaction and decreased the surface area. Several hydrogen bonds were formed between the small molecules.

### 3.6. In Vitro Analysis of ACEI Activity

In this study, DLGGFFGFQR was screened from stout beer using virtual screening and molecular docking. At the next stage, to further verify the actual inhibitory activity of the screened peptide, DLGGFFGFQR was analyzed to identify its in vitro bioactivity. The IC_50_ of DLGGFFGFQR was 24.45 ± 0.35 μM. Previous studies have reported the isolation of numerous ACEI. The ACEI activity of DLGGFFGFQR was more potent than that of VGLFPSRSF [49] and GRVSNCAA [50] and weaker than that of YGIKVGYAIP [51] and DHSTAVW [52]. Activity verification indicated that the peptide obtained via screening methods exhibited significant ACE inhibition effects. Therefore, this study has demonstrated a rapid screening method for ACEI peptides from stout beer. Further in vitro simulation of oral gastrointestinal digestion is necessary to fully understand the action of peptides.

## 4. Conclusions

In this study, stout beer was selected to screen for bioactive peptides using a rapid method. Using this method, 41 peptides were identified, and 6 potential biological activity peptides were selected using in silico tools. Through ADMET prediction and molecular docking, DLGGFFGFQR was assessed to have high ACEI activity. Through exploring the hypotensive mechanisms of DLGGFFGFQR by molecular docking and MD simulation analysis, it was found that the peptide could effectively dock with ACE. Finally, the in vitro activity was verified. This study exhibited a useful method to screen for ACEI peptide with excellent biosafety, thereby providing a theoretical basis for further product innovation and research development of functional beer.

## Figures and Tables

**Table 1:** Peptides of stout beer.

No	Peptide	RT	Tag Length	ALC (%)	Mass	References
1	ALDTRVGV	15.67	8	94	829.4658	
2	LPQQQAQFK	13.86	9	93	1086.5823	Tian et al. [41]
3	STEWHLD	17.12	7	93	886.3821	Tian et al. [41]
4	TDVRLS	10.05	6	93	689.3708	
5	MFALPVPSQPVD	18.84	12	91	1299.6533	
6	VAVAREVAVG	14.41	10	91	969.5607	
7	FDRLQ	11.86	5	91	677.3497	
8	PPPVHD	4.48	6	91	660.3231	Tian et al. [41]
9	LASQLGLGGLSSL	19.07	13	90	1214.6870	
10	QAAVGGQVVEK	12.17	11	90	1084.5876	
11	KELWSMEK	16.11	8	90	1049.5215	
12	TAAGSL	10.38	6	90	518.2700	
13	LVLPGELAK	18.33	9	90	938.5800	Tian et al. [41]
14	EALE	6.20	4	90	460.2169	
15	TALTVVRN	14.20	8	90	872.5079	
16	LAVMQQQQQQ	13.55	10	89	1200.5920	Tian et al. [41]
17	DLGGFFGFQR	18.99	10	89	1142.5508	Picariello et al. [37]
18	SPKMAKNVD	13.08	9	89	988.5012	
19	DNLQGLTKP	15.37	9	89	984.5240	
20	PPPVHDTD	4.46	8	89	876.3977	Tian et al. [41]
21	PPVPHDTD	4.30	8	89	876.3977	Tian et al. [41]
22	ELQTSVR	9.78	7	89	831.4450	
23	LPEDAKVE	13.19	8	88	899.4600	Tian et al. [41]
24	HAVSEGTKAVT	4.73	11	87	1098.5669	Tian et al. [41]
25	SSQGLELSHVFHKS	16.89	14	87	1554.7791	
26	LPGELAK	14.22	7	87	726.4276	Tian et al. [41]
27	AHQQQVPVEVMR	18.09	12	86	1420.7246	
28	PMAPLPRSGEP	15.91	11	86	1150.5803	Picariello et al. [37]
29	TVSGF	15.41	5	86	509.2485	
30	SSLF	18.10	4	86	452.2271	
31	LNFDPNR	15.74	7	86	874.4297	Tian et al. [41]
32	PPPVHDFMNE	4.41	10	86	1181.5176	
33	ELRVR	10.55	5	86	671.4078	
34	EVNNVGQSGLMD	16.34	12	85	1261.5608	
35	ATPCCAEELER	16.22	11	85	1220.5166	
36	VAVARTPTVG	14.57	10	85	969.5607	
37	EAGY	3.79	4	85	438.1750	
38	LALDTRVG	16.39	8	85	843.4814	Tian et al. [41]
39	PMAPLPR	15.47	7	85	780.4316	
40	LDTRVGV	15.14	7	85	758.4286	Tian et al. [41]
41	RVALVY	16.05	6	85	719.4330	Tian et al. [41]

**Table 2:** Peptide Ranker scores of stout beer.

No.	Peptide	Peptide Ranker Score	No.	Peptide	Peptide Ranker Score
1	DLGGFFGFQR	0.8736	21	SPKMAKNVD	0.1683
2	PMAPLPR	0.8565	22	TAAGSL	0.1628
3	SSLF	0.8145	23	LALDTRVG	0.1545
4	PPPVHDFMNE	0.7050	24	LVLPGELAK	0.1488
5	FDRLQ	0.5909	25	VAVARTPTVG	0.1275
6	PMAPLPRSGEP	0.5780	26	EVNNVGQSGLMD	0.1166
7	LASQLGLGGLSSL	0.4581	27	ALDTRVGV	0.1162
8	SSQGLELSHVFHKS	0.4439	28	RVALVY	0.1076
9	PPPVHD	0.4175	29	ELRVR	0.0990
10	PPPVHDTD	0.2941	30	VAVAREVAVG	0.0900
11	STEWHLD	0.2941	31	QAAVGGQVVEK	0.0833
12	TVSGF	0.2924	32	LPEDAKVE	0.0763
13	MFALPVPSQPVD	0.2698	33	LDTRVGV	0.0728
14	ATPCCAEELER	0.2616	34	TDVRLS	0.0723
15	PPVPHDTD	0.2565	35	HAVSEGTKAVT	0.0701
16	AHQQQVPVEVMR	0.2268	36	LAVMQQQQQQ	0.0597
17	LPGELAK	0.2245	37	TALTVVRN	0.0575
18	DNLQGLTKP	0.2083	38	ELQTSVR	0.0521
19	KELWSMEK	0.1888	39	EALE	0.0483
20	EAGY	0.1687			

**Table 3:** Bioactivity prediction of active amino acid sequence of peptide.

No.	Peptide	Activity	Active Amino Acid Sequence
1	DLGGFFGFQR	ACE inhibition	GF, FG, GG, LG, FQ
2	PMAPLPR	ACE inhibition	PLP, PR, PL, AP, MAP
3	SSLF	ACE inhibition	LF
4	PPPVHDFMNE	ACE inhibition	PP, DF, PPP
5	FDRLQ	ACE inhibition	RL, LQ
6	PMAPLPRSGEP	ACE inhibition	PLP, PR, PL, GEP, AP, GE, SG, MAP

**Table 4:** Evaluated properties of stout beer.

9	Peptide	Toxin	GRAVY	Absorption		Excretion
				BBB	HIA	CYP450 Inhibitory Promiscuity
1	DLGGFFGFQR	Non-Toxin	−0.050	0.9493	0.7473	−0.9658
2	PMAPLPR	Non-Toxin	−0.257	0.9332	−0.5054	−0.9958
3	SSLF	Non-Toxin	1.250	0.9037	0.7437	−0.9888
4	PPPVHDFMNE	Non-Toxin	−0.960	0.7896	0.6805	−0.9657
5	FDRLQ	Non-Toxin	−0.980	0.6429	0.7473	−0.9870
6	PMAPLPRSGEP	Non-Toxin	−0.736	0.9399	−0.4929	−0.9953

**Table 5:** Molecular docking score.

Peptide	T-Score	C-Score	Number of Hydrogen Bonds
DLGGFFGFQR	10.96	4.0	9
PPPVHDFMNE	9.22	4.0	5
FDRLQ	8.98	4.0	11

**Table 6:** Action mode of DLGGFFGFQR and ACE.

Peptide	Interaction Mode	ACE Residue
DLGGFFGFQR	Hydrogen bond	Cys352(3.11Å), Gln369(3.00Å), Val380(2.62Å), Pro407(3.09Å), His410(3.06Å), Gly408(2.33Å), Gly414(3.03Å)
	Hydrophobic interaction	Thr166, Lys343, Val148, Thr150, Asp377, Val351, Pro163, Ala149, Gln160, Trp185, Phe512, Ser147, Tyr146, Thr372, Cys370, Lys368, Ile367, Ala381, Leu161, Ser355, Arg522, Ala356, Gly386, Ile413, Ala412, Met385, Ser526

## Data Availability

The original contributions presented in the study are included in the article, further inquiries can be directed to the corresponding authors.
